# OnTheFly: a database of *Drosophila melanogaster* transcription factors and their binding sites

**DOI:** 10.1093/nar/gkt1165

**Published:** 2013-11-22

**Authors:** Shula Shazman, Hunjoong Lee, Yakov Socol, Richard S. Mann, Barry Honig

**Affiliations:** ^1^Howard Hughes Medical Institute, Department of Biochemistry and Molecular Biophysics, Department of Systems Biology, Center for Computational Biology and Bioinformatics, Columbia University, 1130 St. Nicholas Avenue, New York, NY 10032, USA, ^2^Department of Life Science, Open University of Israel, Ra’anana 43107, Israel and ^3^Department of Biochemistry and Molecular Biophysics, Columbia University, 701 West 168th Street, HHSC 1104, New York, NY 10032, USA

## Abstract

We present OnTheFly (http://bhapp.c2b2.columbia.edu/OnTheFly/index.php), a database comprising a systematic collection of transcription factors (TFs) of *Drosophila melanogaster* and their DNA-binding sites. TFs predicted in the *Drosophila melanogaster* genome are annotated and classified and their structures, obtained via experiment or homology models, are provided. All known preferred TF DNA-binding sites obtained from the B1H, DNase I and SELEX methodologies are presented. DNA shape parameters predicted for these sites are obtained from a high throughput server or from crystal structures of protein–DNA complexes where available. An important feature of the database is that all DNA-binding domains and their binding sites are fully annotated in a eukaryote using structural criteria and evolutionary homology. OnTheFly thus provides a comprehensive view of TFs and their binding sites that will be a valuable resource for deciphering non-coding regulatory DNA.

## INTRODUCTION

Specific interactions between transcription factors (TFs) and their DNA binding sites (TFBSs) play a critical role in the control of transcriptional regulation. To decipher the molecular mechanisms underlying these interactions, it is important to collect and analyze known TFs and their corresponding TFBSs. The first studies of TF DNA-binding specificities used biochemical methods such as DNase I footprinting to identify individual binding sites in known target regulatory sequences. Compilation of these sites (1,2) has provided a rich, albeit crude, source of binding-site preferences. Subsequently, a variety of additional methods have been developed to study binding specificities more systematically (3), including systematic evolution of ligands by exponential enrichment (SELEX) (4), SELEX with deep sequencing (5,6) and protein-binding microarrays (PBMs) (7). In addition, the bacterial one-hybrid (B1H) system was developed (8), allowing TF specificities to be determined without the need for protein purification.

Databases that store collections of TF DNA-binding information can be classified by three major criteria (Supplementary Table S1): the species represented in the data set; the type of data stored for each TF (i.e. the sequence or structure of the TF or the TFBS); and the techniques used for collecting the DNA-binding sites (e.g. DNase I or B1H). The commercial database Transfac (2) and the publically accessible database JASPAR (1) include matrix descriptions of recognition motifs for TFs across multiple species. These were generated through a variety of methodologies used to collect the DNA-binding sites, including compiled sequences, B1H, DNase I, SELEX and PBMs. The Uniprobe database provides specificity information for TFs derived from a single technique, PBM, which allows investigators to directly reveal binding site sequence preferences from a diverse collection of organisms including human, mouse and yeast (9).

Several databases focus on TFs encoded in the *Drosophila melanogaster* genome. Of these, FlyBase (10) is the primary database for integrated genetic and genomic data. Information in FlyBase originates from a variety of sources ranging from a large-scale genome projects to the primary research literature. Another *D. melanogaster* TF database is FlyTF (11), which is a manually annotated catalogue of site-specific TFs in the genome. The REDfly database provides an extensive compilation of published experimental data identifying TFBSs (12), while FlyReg (13) comprises a DNase I footprint database and presents a systematic genome annotation of *D. melanogaster* TFBSs. The latter two databases fully merged in 2007 to provide one portal for *D. melanogaster* TFBSs*.* The FlyFactorSurvey (14) database summarizes a project that used the B1H method to systematically describe the binding site preferences of *D. melanogaster* TFs. A smaller database is the Berkeley *D. melanogaster* Transcription Network Project (BDNTP) (15), which focuses on deciphering the transcriptional information contained in the extensive *cis*-acting DNA sequences that control the patterns of gene expression during embryogenesis. Components of this effort include in *vivo* DNA-binding sequences using either the ChIP–chip or the ChIP–seq methods, as well as *in vitro* DNA-binding sequences using the SELEX protocol.

Three-dimensional structural information for TFs and their binding sites in existing databases is limited, although several *D. melanogaster* databases store and present structural annotations for TFs. For example, FlyTF classifies TFs based on the DNA Binding Domains database (DBD) (16). FlyFactorSurvey classifies *D. melanogaster* TFs using Interpro classification (17). Currently, there is no database that contains TF structural models or structural information about the TF-binding sites. Recent studies suggest that an improved understanding of protein–DNA recognition requires that, in addition to the information contained in the linear sequence of nucleotides, DNA shape must also be taken into account (18–21). To integrate sequence and structural information for a single organism, we created OnTheFly (http://bhapp.c2b2.columbia.edu/OnTheFly/index.php), a database for *D. melanogaster* TFs and TFBSs. OnTheFly currently houses DNA recognition motifs for >387 genes encoding TFs (>50% of the predicted *Drosophila* TF genes), and it extracts binding sites based on multiple data sources (e.g. DNase I, B1H and SELEX). OnTheFly also provides structural information for both TFs and their binding sites whenever possible. We believe that the scope of its coverage and its integration of both sequence and structural information renders it as an important tool in the study of the interactions between TFs and their DNA-binding sites.

## MATERIALS AND METHODS

### Annotating and classifying *D. melanogaster* TFs

A list of 2107 *D. melanogaster* candidate TFs encoded by 754 genes (the 754 genes encode 2107 splice isoforms) was extracted from Ensembl (release version 71; http://ensembl.org/), based on the protocol described in FlyTF (11,22). Specifically, a TF is chosen based on either the presence of a canonical DNA-binding domain predicted with the DBD database (16) or based on direct experimental evidence. The list of TFs is composed of 1970 proteins that possess canonical DNA binding domains and 137 that do not. TFs were classified based on the domains they possess that are defined in Interpro in a hierarchical fashion. For example, an Interpro entry might represent a subclass of a broad class of domains that share structure and/or function. On this basis of the 113 different Interpro entries represented in *Drosophila*, the TFs were grouped into 18 sets of DNA-binding domains that each include at least 10 TFs (OnTheFly Domain Name; see Supplementary Table S2). A 19th category, ‘Other’, contains Interpro entries with <10 TFs. We used Interpro (17) for classification because it integrates domain annotations based on 12 different methods including those used in DBD (16). We found 120 additional DNA-binding domains in Interpro that do not appear in DBD (see Supplementary Table S3 for examples).

### TF structures

OnTheFly provides either experimentally derived structures or homology models for most (74%) of the TFs in the database. Experimental structures were obtained by querying the PDB using Protein KnowledgeBase (UniProtKB) accession numbers. Protein structures or protein–DNA complexes (X-Ray or NMR) were found for 65 of the *D. melanogaster* TFs; these structures were linked to OnTheFly. In cases where a TF was included in more than one structure, all relevant links to the PDB were included. For TFs for which experimental structures were not available, a search for homology models was conducted using the Modbase database (23), which was queried with UniProt accession numbers. Homology models were found in Modbase for 1171 of the *D. melanogaster* TFs and stored in OnTheFly.

Homology models in Modbase all have e-values < 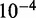. To expand our structural coverage to TFs not in Modbase, homology models were constructed with the PUDGE homology modelling pipeline (24) using HHPRED 1.5 (25) for template selection (homology models were built only where e-values for template selection were < 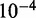), MODELLER for model building (26) and the pG score derived from PROSA-II (27,28) for model evaluation. Homology models were stored in OnTheFly only when the pG score was > 0.5. Using PUDGE, 318 homology models with an e-value < 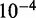 and a pG score > 0.5 were added to OnTheFly.

### DNA shape parameters

When experimentally derived structural information (X-ray or NMR) on protein–DNA complexes was available, minor groove width, roll, propeller twist and helix twist were measured along the DNA sequence using CURVES 5.1 (29) and stored in the database (see example in Supplementary Figure S2). In addition, for all cases where Position Weighted Matrices (PWMs) were available, DNA shape parameters are provided via a link to a web server that predicts DNA structural features using a high-throughput (HT) method based on Monte Carlo simulations (30). Currently, the database represents the predicted DNA shape parameters for all DNA sequences that contributed to the PWM.

## DATABASE CONTENT

OnTheFly annotates 2107 proteins derived from 754 genes. TF structures were obtained from the PDB (65 TFs) and homology models (1489 TFs, 1171 from Modbase and 318 using the PUDGE homology modelling pipeline). Inferred motifs of TFBSs are presented in the database using a PWM, and were obtained from several sources: 87 PWMs based on DNaseI footprint data were extracted from FlyReg (13); 327 PWMs based on B1H were extracted from FlyFactorSurvey (14); 22 PWMs based on SELEX data were extracted from a study of Hox proteins (6), from BDNTP (15) and from JASPAR (1). Taken together, OnTheFly houses DNA recognition motifs for >387 different genes encoding TFs (>50% of the genes), comprising the largest collection of TFBS recognition motifs currently available for *D. melanogaster*. The DNA recognition motifs in OnTheFly are organized by TF although in several cases where a PWM was connected with a gene and not with a TF, all gene isoforms are linked to the same PWM.

**Figure 1.**
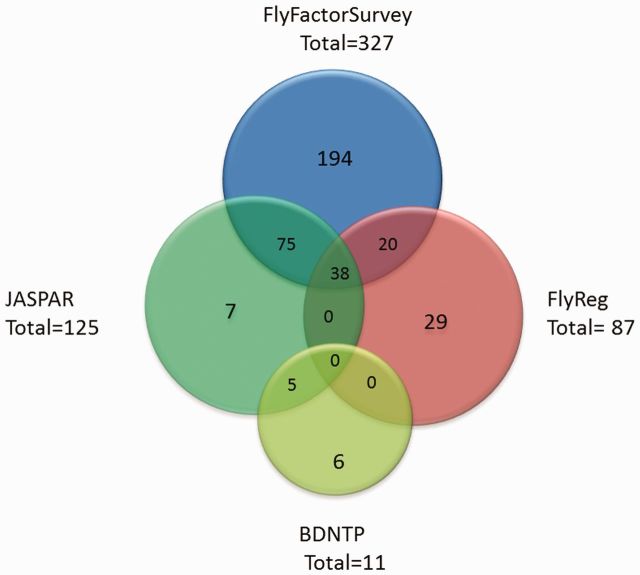
The contribution of previous databases to the PWMs appearing in OnTheFly.

Figure 1 displays a Venn diagram reporting the contribution of the different databases to the PWMs collected in OnTheFly. As is evident, the largest contribution is from B1H data stored in FlyFactorSurvey (327 genes; 43% of all *Drosophila* TF genes), with smaller contributions coming from JASPAR, FlyReg and BDNTP. Combining the PWM motifs from all databases, OnTheFly includes PWMs for 387 genes; 51% of all *Drosophila* TF genes.

The distribution of TFs among different structural families is shown in Supplementary Figure S1A. TFs with multiple DNA-binding domains are classified by each of their respective families, whereas TF families with <10 members are classified as ‘other’. The Classical Zinc Finger (C2H2 and C2CH) family contains ∼700 TFs, about a third of all *D. melanogaster* TFs, and ∼300 TFs possess a homeodomain (encoded by 436 and 138 genes, respectively). As shown in Supplementary Figure S1B, the majority of *D. melanogaster* TFs possess a single DNA-binding domain, whereas 8% of all TFs possess two DNA-binding domains from different structural families. TFs possessing DNA-binding domains from three or more different structural families were not found. The combinations of DBD pairs are shown in Supplementary Figure S1C. Supplementary Figure S1D describes the number of TFs and genes encoding TFs from each of the DNA binding domain families for which a PWM is known. As shown in Supplementary Figure S1D, the homeodomain family has the largest number of known PWMs.

## WEB INTERFACE

### Database organization

All the information in OnTheFly is stored with MySQL, a free database management system widely used in bioinformatics.

### Data searching

**Figure 2.**
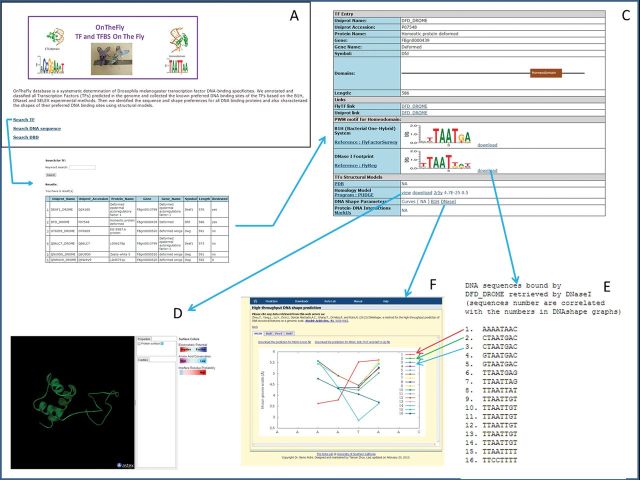
TF search workflow in OnTheFly. This figure describes a search for a sequence-specific transcription factor, *Homeotic protein Deformed* (*DFD*). (**A**) In the Entry Screen, we choose *TF search*. (**B**) A search for the term *Deformed* retrieves six TFs. (**C**) Choosing the second, *DFD_DROME*, leads to a detailed TF screen. This screen shows that DFD possesses a homeodomain and has two known TFBS represented by a PWM, one based on B1H data and the other based on DNase I data. (**D**) A homology model for this protein shows three alpha helices comprising the homeodomain shown using the MarkUs viewer. (**E**) The DNA sequences retrieved by DNase I are sorted according to their putative binding affinity to this protein. (**F**) Opening the DNaseI or B1H links shows the results of the DNA shape server (30). Each line in the graph represents the minor groove width along a different DNA sequence, which was entered as input. The graph shows that most of the sequences possess a minimum in width (narrower minor groove width in the *AT* part of the DNA sequence motif).

OnTheFly provides three different approaches for data searching: by TF, by DNA sequence and by DBD. Figure 2 shows a schematic workflow for a sample TF search. Movie S1 shows the search process by DNA sequence. PWMs are linked to 18 sets of Interpro DNA-binding domains to allow users to find PWMs for specific DNA-binding domains (see Supplementary Figure S2).

### MarkUs function-annotation server

The MarkUs server (31) integrates various sequence- and structure-based analysis tools to characterize the biochemical and biophysical properties of a protein structure and identifies structural neighbors as a basis of function annotation. The interface enables the selection and display of functional information associated with structural neighbours of the query protein. Overall annotations of a protein (GO term, EC class) and annotations associated with individual residues (UniProt sequence features, ligand interactions) can be displayed and used to filter structural neighbours to create subsets of functionally related proteins. Functional properties of a structural neighbour can also be visualized in the query structure itself using the AstexViewer 2.0^1^. MarkUs allows the user to examine the query protein for properties such as electrostatic potentials, solvent accessible cavities, interfacial residues, domain information and amino acid conservation.

Protein structures, protein–DNA complexes and DNA structures can be visualized with MarkUS. Two types of representations are available for the display of DNA structures using either line representations or the molecular surfaces with convex regions coloured in green and concave regions coloured in gray. This type of curvature representation provides users a clear picture of major and minor groove shapes.

## CONCLUSIONS

*D. melanogaster* is an important model organism, and its genome encodes numerous members of all known families of DNA-binding proteins. In the OnTheFly database, PWM motifs of DNA-binding sites are available for >50% of the genes encoding TFs in this organism, a relatively high percentage compared with other TF databases or known PWM datasets for other species [e.g. human (5) and mouse (32)]. OnTheFly is designed to annotate all DNA-binding TFs and their binding specificities and to assemble available sequence and structural information for all TFs encoded in the *D. melanogaster* genome, as well as their binding sites. OnTheFly can thus be of use for various applications such as studying interactions between TFs and DNA, predicting the most likely specific DNA sequence recognized by a novel TF or predicting the potential interactions between a TF and a specific DNA sequence, based on various DNA structural parameters.

OnTheFly will continue to be regularly updated as new structural and PWM data become available. In the coming year, the database will also be expanded to include PWMs for orthologs of *Drosophila* TFs (human, mouse and yeast) that are retrieved by PBM, B1H or SELEX methods. Whenever available, OnTheFly will also be expanded to increase the structural coverage of TFs and new information about DNA structure derived from improved simulations.

## SUPPLEMENTARY DATA

Supplementary Data are available at NAR Online.

## FUNDING

Funding for open access charge: National Institutes of Health [U54-CA121852 and RO1-GM054510].

*Conflict of interest statement*. None declared.
